# Oscillations of the circadian clock protein, BMAL-1, align to daily cycles of mechanical stimuli: a novel means to integrate biological time within predictive in vitro model systems

**DOI:** 10.1007/s44164-022-00032-x

**Published:** 2022-08-31

**Authors:** Hannah K. Heywood, Laurence Gardner, Martin M. Knight, David A. Lee

**Affiliations:** 1 School of Engineering and Materials Science, Queen Mary University of London, London, UK; 2 Wirral University Teaching Hospital NHS Foundation Trust, Liverpool, UK

**Keywords:** Circadian clock, Mechanobiology, Organ-on-a-chip, Predictive in vitro model

## Abstract

**Purpose:**

In vivo, the circadian clock drives 24-h rhythms in human physiology. Isolated cells in vitro retain a functional clockwork but lack necessary timing cues resulting in the rapid loss of tissue-level circadian rhythms. This study tests the hypothesis that repeated daily mechanical stimulation acts as a timing cue for the circadian clockwork. The delineation and integration of circadian timing cues into predictive in vitro model systems, including organ-on-a-chip (OOAC) devices, represent a novel concept that introduces a key component of in vivo physiology into predictive in vitro model systems.

**Methods:**

Quiescent bovine chondrocytes were entrained for 3 days by daily 12-h bouts of cyclic biaxial tensile strain (10%, 0.33 Hz, Flexcell) before sampling during free-running conditions. The core clock protein, BMAL-1, was quantified from normalised Western Blot signal intensity and the temporal oscillations characterised by Cosinor linear fit with 24-h period.

**Results:**

Following entrainment, the cell-autonomous oscillations of the molecular clock protein, BMAL-1, exhibited circadian (24 h) periodicity (*p* < 0.001) which aligned to the diurnal mechanical stimuli. A 6-h phase shift in the mechanical entrainment protocol resulted in an equivalent shift of the circadian clockwork. Thus, repeated daily mechanical stimuli synchronised circadian rhythmicity of chondrocytes in vitro.

**Conclusion:**

This work demonstrates that daily mechanical stimulation can act as a timing cue that is sufficient to entrain the peripheral circadian clock in vitro. This discovery may be exploited to induce and sustain circadian physiology within into predictive in vitro model systems, including OOAC systems. Integration of the circadian clock within these systems will enhance their potential to accurately recapitulate human diurnal physiology and hence augment their predictive value as drug testing platforms and as realistic models of human (patho)physiology.

**Supplementary Information:**

The online version contains supplementary material available at 10.1007/s44164-022-00032-x.

## Introduction

In vivo, the circadian system plays an overarching role in regulating human physiology [1–3]. A cellular timing system, or circadian clock, is present in all our tissues which drives near-24-h rhythms in transcription [1, 4, 5]. Up to 20% of a tissue’s proteome is regulated by the circadian clock [6, 7]. This co-ordinates the temporal compartmentalisation of vital cellular processes to anticipate the differing demands of the day or night, generating time-of-day specific cellular physiology [8–10]. For example, the O’Neill lab has demonstrated that the circadian clock regulates actin dependent processes such as cell migration and adhesion, which ultimately creates remarkable time-of-day differences in wound healing efficiency [9].

In vivo, the central clock within the suprachiasmatic nucleus (SCN) within the brain aligns to light–dark cycles and in turn synchronises the clocks within peripheral tissues via systemic signals including corticosteroids and temperature cycles [11, 12]. Peripheral clocks also align to other pertinent environmental stimuli, known as Zeitgebers (“time-giver”), in a process called entrainment [5, 13]. The molecular clock mechanism consists of core clock genes, CLOCK, BMAL1, cryptochrome (CRY), period (PER), and their regulators. Isolated cells retain a functional clockwork but lack the necessary timing cues experienced in vivo, so that the cell-autonomous clock within cultured cells become unsynchronised, leading to the rapid loss of tissue-level circadian rhythms [14, 15]. Therefore, the direct entrainment of peripheral clocks by local timing cues is an understudied but important area.

**Fig. 1:**
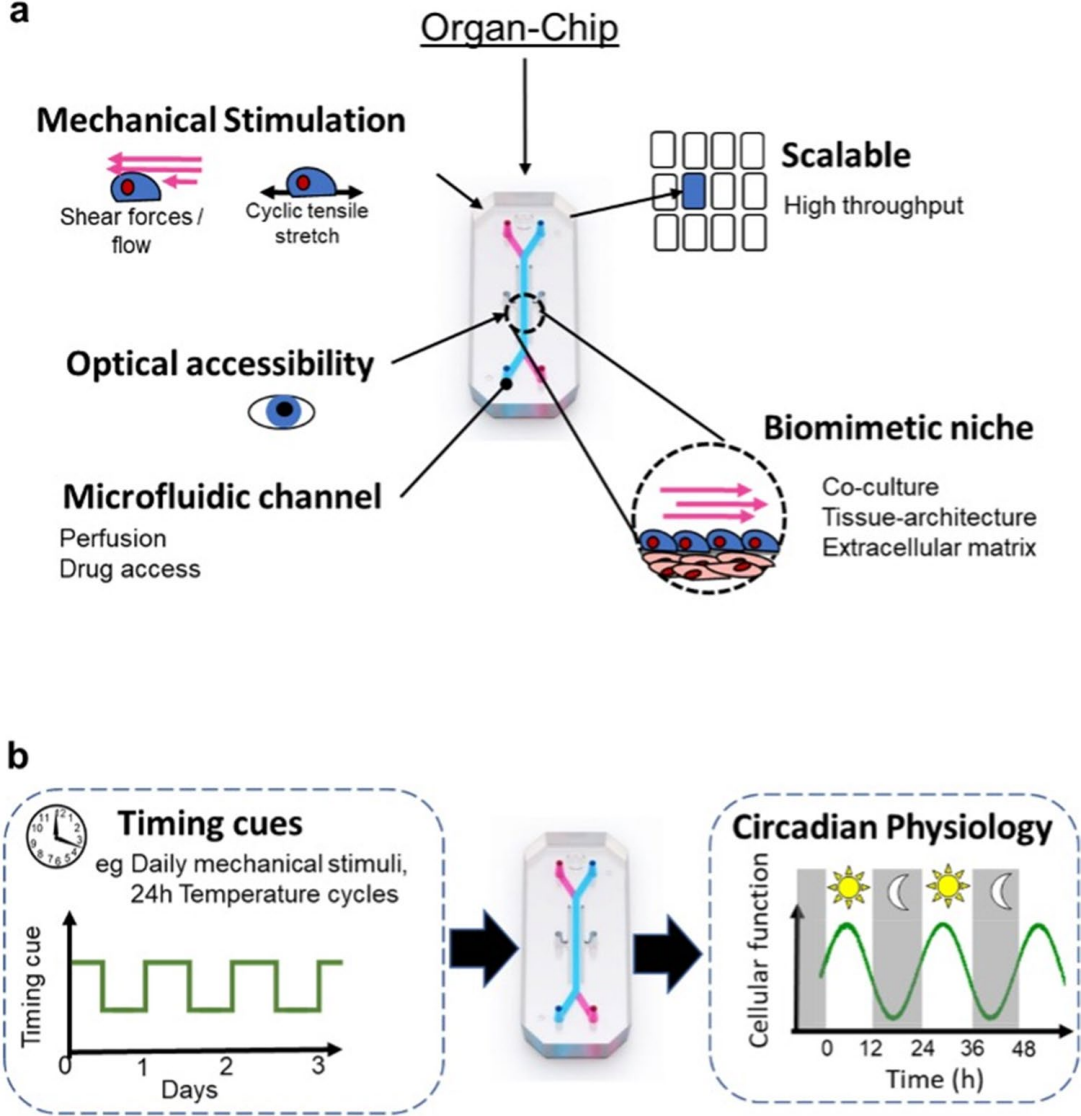
**a** Schematic illustrating features of a typical organ-on-a-chip system, which aim to recapitulate the cellular niche in vivo. However current predictive in vitro models including such OOAC systems do not incorporate the concept of biological “time-of-day,” due to the absence of relevant timing cues to synchronise the peripheral circadian clock. **b** We propose that daily patterns of mechanical stimuli can act as a timing cues for the peripheral circadian clock and may therefore be exploited to simulate diurnal human physiology within in vitro systems to augment their predictive value as drug testing platforms and as realistic models of human disease. Image of organ-chip courtesy of Emulate Inc

The limitations of traditional 2D in vitro cell cultures and animal models have inspired the development of complex predictive in vitro model systems, including human organ-on-a-chip (OOAC) systems that aim to represent the minimally functional unit of target organs. These systems aim to mimic organ level physiology by incorporating features such as 3D culture, co-culture of multiple cell types, and appropriate physiological mechanical stimuli such as flow and stretch [16, 17] (Fig. 1a). Predictive in vitro models promise to accelerate research into human physiology and have potential ultimately to replace animal models in pre-clinical therapeutic testing. The accurate recapitulation of target organ physiology is therefore an essential determinant of the value of the model system. However, current technologies do not replicate circadian rhythmicity and this limits their predictive value in situations where the target physiology has a circadian component. The incorporation of relevant timing cues into predictive in vitro model systems is a concept that has received limited attention to date, in part due to the technical challenges of recapitulating the circadian profile of synchronising endocrine signals such as cortisol within these systems [18, 19]. However, the application of appropriate timing cues represents an important area of development to introduce a key component of in vivo physiology into OOAC technology and other predictive in vitro systems (Fig. 1b).

Cells are highly mechanosensitive and express multiple mechanosensors [20] that enable them to discriminate between different magnitudes, modes, and frequencies of mechanical stimuli, such as stretch and shear forces that themselves vary between day and night. OOAC technologies typically incorporate physiological mechanical stimulation but fail to acknowledge the diurnal pattern of such stimuli in vivo. Recent studies have revealed interactions between the biomechanical environment and clock function. Our analysis of the RNAi screen for clock modulators (BioGPS.org database) reported by Zhang and colleagues [21] finds altered amplitude of clock oscillations following mechanoreceptor siRNA knockdown, as does altered substrate stiffness [22]. However, it is not known whether mechanical stimuli can phase shift, or “set the time,” of the peripheral circadian clock. The current project therefore aims to reveal whether diurnal mechanical stimuli represents a novel timing cue that synchronises mechanosensitive cells in vitro. The findings are highly relevant to the fields of chronobiology and development of highly predictive pre-clinical in vitro models.

## Materials and methods

### Cell source, culture, and entrainment

**Fig. 2:**
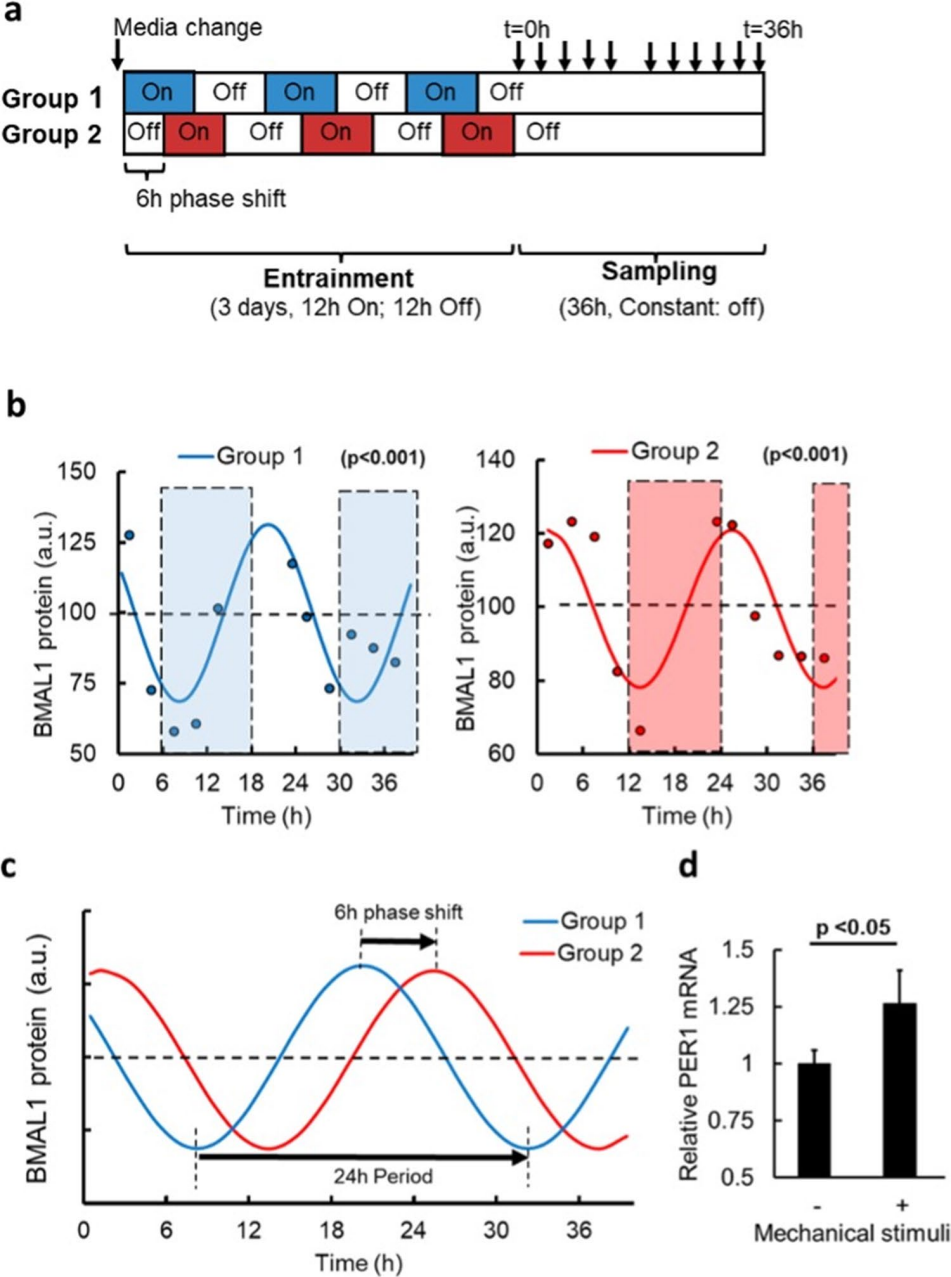
Circadian oscillations of the clock protein, Bmal-1, align to daily patterns of mechanical stimuli **a** Schematic of the entrainment protocol by daily patterns of mechanical stimulation and subsequent sampling time-course under constant conditions. Cyclic biaxial stretch (10%, 0.33 Hz) was applied for 12 h alternating with 12 h rest for 3 days, to entrain the circadian clock, before sampling during constant unloaded conditions. Group 1 (red) and group 2 (blue) cultures where cultured and sampled in parallel, but group 2 had a 6 h phase delay or “jet lag” in the entrainment protocol. **b** Quantification of the circadian clock protein, BMAL-1, from normalised Western blot signal intensity, showing that cell autonomous oscillations of BMAL-1 protein align to daily patterns of mechanical stimuli. A linear model was fit to the data points of each group using Cosinor package (R studio) with 24 h periodicity (*p* < 0.001). There were 11 datapoints per time series illustrated, with replicate timeseries yielding comparable results. The shaded areas on the graphs illustrate times of mechanical stimulation anticipated for each group, according to the prior loading protocol shown in **a**. The cell autonomous circadian oscillations in BMAL-1 demonstrated a nadir in the early anticipated loaded phase and peak in the early anticipated rest phase. **c** Overlay comparison of the cosinor linear models from B shows a 6-h phase delay in clock oscillations in response to a 6-h shift in loading protocol. **d** Array data illustrating mechanosensitive Per1 gene expression in response to a single 5 h bout of mechanical stimuli (*n* = 3). Together, these data demonstrate that daily patterns of mechanical stimuli acts as a novel timing cue that is sufficient to re-set the circadian clock within in vitro systems

Primary chondrocytes were enzymatically isolated from bovine (a diurnal species) articular cartilage [23] and seeded at a near confluent density of 8 × 10^4^ cells/cm^2^ onto silicone 6-well-plates (bioflex, Flexcell) with 3 mL DMEM + 10% serum. The plates were pre-coated with type I collagen to promote adhesion and loaded into Flexcell mechanical stimulation apparatus within a cell culture incubator. The Flexcell apparatus is illustrated schematically in Fig. 1 of Bleuel et al.[24]. After 4 days, the media was completely replenished in all samples. Entrainment to mechanical stimuli was achieved by exposing the quiescent cultures to biaxial cyclic tensile stretch (10%, 0.33 Hz), for 12 h alternating with 12-h rest for a total of 3 days. Two experimental groups followed an identical preparation, feeding schedule, entrainment and sampling protocol, with the exception that a 6-h delay or “jet lag” was applied to the mechanical stimulation of group 2 relative to group 1, illustrated schematically in Fig. 2a. The parameters 10% and 0.33 Hz cyclic stretch were selected as chondrocytes are demonstrated to exhibit an anabolic mechanotranduction response to this regime [24, 25] and this also falls within the typical range achieved by OOAC devices.

### Sampling protocol, protein extraction, and quantification

The expression of the molecular clock protein, BMAL-1, during the 36 h constant (unloaded) conditions following entrainment by mechanical stimuli was quantified by Western blot. Protein samples were collected at approximately 3-h intervals, illustrated in Fig. 2a. For each sample, the cells from two wells were rinsed with PBS followed by scraping and pooling into 100 μL lysis buffer (RIPA, Sigma + protease inhibitor cocktail, Roche) and kept on ice for 10 min before snap freezing on dry ice and storage at − 80 °C. Duplicate samples were collected per timepoint. On completion of the timecourse, all the samples were simultaneously de-frosted and sonicated for 3 × 10 s prior to centrifuge at 14,000 g for 15 min at 4 °C and the protein concentration of the supernatant quantified by BCA assay (Pierce). The protein concentration was equalised using lysis buffer, combined in 3:1 ratio with 4 × Laemmli buffer + 10% 2-merceptoethanol and heat-denatured for 5 min.

### Gel electrophoresis and Western blotting for BMAL-1

An equal quantity of protein in 16 μL was loaded per well of pre-cast mini-protean TGX gels 4–20% gels (Biorad), with one gel per timeseries. Electrophoresis was applied according to the manufacturers protocol with a tris–glycine-SDS running buffer (Biorad). Protein transfer onto nitrocellulose membranes (Biorad) was performed using the trans-blot turbo mini kit (Biorad). The membranes were allowed to dry at room temp for 1 h prior to quantifying the total protein using the Li-Cor total protein stain protocol and Odyssey blot-imager (Li-Cor). The membranes were blocked using 5% non-fat milk powder (Marvel) in Tris-buffered saline (TBS) for 1 h at room temperature before incubating overnight at 4 °C with primary antibody (rabbit anti-bmal-1, abcam ab93806) diluted 1:3000 in 2.5% milk in TBS + 0.1% tween-20. After washing, membranes were incubated for 1 h at room temp with secondary antibody, donkey anti-rabbit IRDye 800CW (Licor, 925–32,213) diluted 1:10,000 in TBS + 0.2% tween-20. The membranes were washed extensively before imaging with the Li-Cor Odyssey blot-imager. The 800 nm fluorescence signal from BMAL-1 was quantified using the Image Studio Lite software (Li-Cor Bioscience) and normalised to total protein. Representative Western blot images are presented in Fig. S1 in the supplementary material.

### PER1 gene expression

To demonstrate if the core clock gene PER1 gene expression is mechanosensitive, unsynchronised monolayer cultures of chondrocytes were exposed to a single 5-h episode of mechanical stimuli from hyperosmotic (500 mOsm) media exposure, and PER1 mRNA was quantified by qPCR micro-array. Chondrocytes were seeded in 6-well plates (VWR), with a seeding density of 5 × 10^4^ cells/cm^2^ and cultured overnight. Subsequently, the specimens were subjected to mechanical stimuli, involving osmotic challenge at 500 mOsm/kg for 5 h, with control samples maintained at 300 mOsm/kg. The total RNA from each specimen was extracted directly after the osmotic challenge regime, using an RNA extraction kit (RNeasy Mini Kit, Qiagen, Crawley, UK). The cDNA and cRNA were synthesised from the total RNA by using Illumina TotalPrep RNA Amplification Kit (Ambion, Paisley, UK). The cRNA samples were hybridised onto MouseRef-8 v2.0 Expression BeadChips (Illumina), stained with Cy3- streptavidin, and the genome wide expression level was measured by Illumina iScan (Illumina, Little Chesterford, UK). The MouseRef-8 v2.0 BeadChip contains 8 arrays, each of which contains 25,697 probes that represent 19,100 genes in total. Data for PER1 were extracted from the array dataset and statistical comparisons with unloaded control (300 mOsm) samples were by *t*-test (*n* = 3).

### Statistical analysis

A model with 24-h periodicity was fit to the 11 data points of each BMAL-1 protein timeseries using the Cosinor package within the R-studio platform [26].

## Results

Cells were entrained for 3 days by daily mechanical stimuli before sampling at intervals under constant free-running conditions, illustrated schematically in Fig. 2a. Oscillations of the molecular clock protein, BMAL-1, were observed during the free-running (unloaded) conditions following entrainment by mechanical stimuli (Fig. 2b). This demonstrates the cell-autonomous nature of the clockwork and its synchronisation within the cell population in vitro. A linear model was fit to the timeseries from group 1 and group 2 using the Cosinor package (R studio) with 24-h periodicity (*p* < 0.001), indicating the rhythmicity was circadian (Fig. 2b).

In a novel discovery, it was observed that the oscillations in BMAL-1 aligned to the diurnal patterns of mechanical stimuli. The shaded regions in Fig. 2b illustrate the times that mechanical stimulation would be anticipated and had the prior loading protocol shown in Fig. 2a been continued into the sampling phase. In each group, the cell autonomous oscillations in BMAL-1 demonstrated a nadir in the early anticipated loaded phase and peak in the early anticipated rest phase. Like other tissues, cartilage experiences varying intensity of mechanical stimuli in vivo, with higher loading during the daytime active phase in diurnal species such as man, and relative unloaded conditions during rest at night. Therefore, the shaded areas in Fig. 2b are akin to the subjective active phase and the unshaded to the subjective rest phase. Remarkably, this finding is in keeping with the findings of Mure and colleagues [1], who report that peak expression of BMAL-1 in the peripheral clock of a diurnal species aligns with transition from light to dark.

Next, it was examined whether a 6-h shift in the mechanical stimulation protocol elicited an equivalent phase shift in the BMAL-1 oscillation. Group 1 (red) and group 2 (blue) cultures where isolated, cultured, fed, and sampled in parallel, but group 2 had a 6-h delay or “jet lag” in the mechanical stimuli during the entrainment protocol. The Cosinor linear models from Fig. 2b are overlaid in Fig. 2c to illustrate the observed 6-h phase delay of circadian oscillations in group 2 relative to group 1. This is the first evidence demonstrating that cells can directly sense the daily pattern of mechanical stimuli and align their circadian clockwork (and hence their clock-controlled physiology) to it. Thus, rather than being simply responsive to mechanical stimuli, cells are able sense daily rhythms in mechanical stimuli and align their physiology in anticipation of it.

To examine if other genes of the circadian clockwork are mechanosensitive, the expression of PER1 gene was examined by PCR array in unsynchronised chondrocytes following a single episode of mechanical stimuli. A significant upregulation of PER1 gene expression relative to unstimulated controls was observed (*p* < 0.05, *t*-test). This observation is pertinent as PER1 is not only essential to clock rhythmicity but is also reported to be an early response gene to other, established, entrainment stimuli [27].

Together, these data demonstrate that daily cycles of mechanical stimuli are a timing cue, or zeitgeber, for the peripheral circadian clock mechanism, which is sufficient to synchronise the cellular clock within in vitro culture systems. Furthermore, this data supports the concept that daily patterning of mechanical stimuli may replicate in vitro the active:rest phases of the circadian cycle that occur in vivo.

## Discussion

The data presented in this study demonstrate that the circadian clockwork within isolated chondrocyte cells aligns to daily patterns of mechanical stimuli. This indicates that mechanical stimuli can act as a direct entrainment factor (timing cue) for the peripheral clock, which is an important yet understudied area of chronobiology. The implications of mechanical stimuli as a novel entrainment factor are broad-reaching: Repeated daily mechanical stimuli have potential to synchronise cell populations to “set the time” in the tissue; once entrained, cellular physiology pre-emptively adapts in *anticipation* of daily mechanical stimuli, rather than being simply responsive to it. We propose that the introduction of circadian timing cues, including daily patterning of mechanical stimuli illustrated in this study, will sustain circadian biology in vitro, thus enabling in vitro systems to better mimic in vivo diurnal human physiology. Accurately replicating diurnal physiology in vitro has the potential to transform the predictive potential of OOAC systems for research and pre-clinical testing.

The current study utilised isolated chondrocytes as an exemplar model system. The cartilage circadian clock drives 24-h rhythmic gene expression such that a considerable proportion of the cartilage proteome (12.3%) in vivo exhibits 24-h rhythms in abundance [7, 28]. Accordingly, cellular physiology in vivo differs markedly according to the phase of the clock, i.e., “time of-day.” The temporal segregation of key physiological functions by the circadian clock is also exemplified in numerous other tissues, with up to 20% of the proteome subject to circadian control [6]. This can result in dramatic time-of-day specific cellular behaviour, attributable to the circadian clock. For example, Hoyle and colleagues [9] found that in humans, daytime wounds healed ~ 60% faster than night-time wounds, attributed to cell-intrinsic rhythms in cytoskeletal actin dynamics. In another example, Zhang and colleagues [29] report that drug efficacy may differ 2–tenfold depending on the time of day, due to circadian rhythmicity of the drug target. This highlights the importance of incorporating circadian timekeeping within in vitro models across numerous tissue types and applications.

The majority of WHO essential medicines may benefit from timed administration or “Chronotherapy,” based on circadian rhythmicity of the drug target and relatively short drug half-life [29]. The benefits of timed dosage for a very limited number of individual drugs have been confirmed in clinical trials [30, 31]. Yet, there is no platform incorporating human circadian rhythmicity for research and validation prior to clinical use, which would enhance drug discovery programmes. This project demonstrates the potential to utilise mechanical stimuli to induce and sustain circadian rhythmicity within OOAC technology that may in turn replicate human circadian physiology. This represents a novel research tool to evaluate investigative therapies and disease models in the presence of circadian control. This tool may also be used to examine conditions that lead to disruption of normal clock function, a causal factor in many diseases, including osteoarthritis and arteriosclerosis [32–35].

The current data demonstrates control of the cartilage peripheral clock by mechanical stimuli, but this exciting novel concept demands further research to fully capitalise on this discovery. Further studies are needed demonstrate the subsequent regulation of tissue specific clock-controlled physiology following mechanical entrainment. Future work should also aim to establish whether mechanical stimuli represents a universal entrainment factor, common to all peripheral tissue types, as was reported of 24-h temperature cycles and corticosteroid pulses [11, 12], to determine the breadth of the relevance, as well as the hierarchy and synergy of this novel timing cue in combination with established timing cues. Earlier work by our group has also shown that the anabolic response of chondrocytes to 12 h:12 h on:off repeated daily bouts of cyclic mechanical stimuli is greater than alternative intermittent or continuous loading regimes with equivalent number of loading duty cycles [36]. It would be interesting to determine whether the circadian clock mechanism is essential to this observation, which could indicate “time-of-day” specificity of the mechanoresponse, in addition to mechanical regulation of the clock illustrated in this study.

Next steps also include elucidating the signalling pathways that are involved in transducing mechanical signals to the circadian clock. The CREB (cyclic AMP response element binding protein)/CRE (cyclic AMP response element) pathway is a known signalling conduit coupling timing cues to the resetting of the circadian clockwork [37, 38]. Upon phosphorylation of CREB, CRE-mediated transcriptional induction of the core clock genes, Period 1 and Period 2, initiates phase resetting of the circadian clockwork [39–41]. Furthermore, the CREB/CRE pathway can regulate the amplitude of the core clock timing rhythm [42]. The present study indicates Per1 as an early mechanosensitive gene and it has been shown by our lab (data not shown) and others [43] that CREB is phosphorylated upon mechanical stimulation; therefore, the role of the CREB/CRE pathway in the transduction of mechanical signalling to the circadian clock mechanism warrants further investigation.

## Conclusion

This study shows mechanical stimuli can act as a direct timing cue for the peripheral circadian clock that is sufficient to set the time in isolated cells. These findings may be exploited to synchronise the peripheral circadian clock and sustain circadian physiology within predictive in vitro model systems. Introducing the circadian clock within in vitro models such as OOAC systems would enhance their potential predictive value as drug testing platforms and models of human (patho)physiology. Enhancing the predictive power of OOAC technology in this way may, in turn, ultimately enhance the success rate of costly clinical trials which currently have low efficiency.

## Supplementary Information

Below is the link to the electronic supplementary material.

Supplementary file1 (PDF 1059 KB)

## Data Availability

The datasets generated during and/or analysed during the current study are available from the corresponding author on reasonable request.

## References

[1] Mure LS, Le HD, Benegiamo G, Chang MW, Rios L, Jillani N, et al. Diurnal transcriptome atlas of a primate across major neural and peripheral tissues. Science. 2018;359(6381):eaao0318. 10.1126/science.aao0318. 10.1126/science.aao0318 PMC5924732 29439024

[2] Yeung J, Naef F. Rhythms of the genome: circadian dynamics from chromatin topology, tissue-specific gene expression, to behavior. Trends Genet. 2018;34(12):915–26. 30309754 10.1016/j.tig.2018.09.005

[3] Ruben MD, Wu G, Smith DF, Schmidt RE, Francey LJ, Lee YY, et al. A database of tissue-specific rhythmically expressed human genes has potential applications in circadian medicine. Sci Transl Med. 2018;10(458):eaat8806. 10.1126/scitranslmed.aat8806. 10.1126/scitranslmed.aat8806 PMC8961342 30209245

[4] Anafi RC, Francey LJ, Hogenesch JB, Kim J. CYCLOPS reveals human transcriptional rhythms in health and disease. Proc Natl Acad Sci. 2017;114(20):5312–7. 28439010 10.1073/pnas.1619320114 PMC5441789

[5] Dibner C, Schibler U, Albrecht U. The mammalian circadian timing system: organization and coordination of central and peripheral clocks. Annu Rev Physiol. 2010;72(1):517–49. 20148687 10.1146/annurev-physiol-021909-135821

[6] Reddy AB, Karp NA, Maywood ES, Sage EA, Deery M, O’Neill JS, et al. Circadian orchestration of the hepatic proteome. Curr Biol. 2006;16(11):1107–15. 16753565 10.1016/j.cub.2006.04.026

[7] Dudek M, Angelucci C, Pathiranage D, Wang P, Mallikarjun V, Lawless C, et al. Circadian time series proteomics reveals daily dynamics in cartilage physiology. Osteoarthr Cartil. 2021;29(5):739–49. 10.1016/j.joca.2021.02.008 PMC8113022 33610821

[8] Chang J, Garva R, Pickard A, Yeung C-YC, Mallikarjun V, Swift J, et al. Circadian control of the secretory pathway maintains collagen homeostasis. Nat Cell Biol. 2020;22(1):74–86. 10.1038/s41556-019-0441-z PMC7613259 31907414

[9] Hoyle NP, Seinkmane E, Putker M, Feeney KA, Krogager TP, Chesham JE, et al. Circadian actin dynamics drive rhythmic fibroblast mobilization during wound healing. Sci Transl Med. 2017;9(415):eaal2774. 10.1126/scitranslmed.aal2774. 10.1126/scitranslmed.aal2774 PMC5837001 29118260

[10] Hayter EA, Wehrens SMT, Van Dongen HPA, Stangherlin A, Gaddameedhi S, Crooks E, et al. Distinct circadian mechanisms govern cardiac rhythms and susceptibility to arrhythmia. Nat Commun. 2021;12(1):2472. 33931651 10.1038/s41467-021-22788-8 PMC8087694

[11] Buhr ED, Yoo S-H, Takahashi JS. Temperature as a universal resetting cue for mammalian circadian oscillators. Science. 2010;330(6002):379–85. 20947768 10.1126/science.1195262 PMC3625727

[12] Balsalobre A, Brown SA, Marcacci L, Tronche F, Kellendonk C, Reichardt HM, et al. Resetting of circadian time in peripheral tissues by glucocorticoid signaling. Science. 2000;289(5488):2344–7. 11009419 10.1126/science.289.5488.2344

[13] Stokkan K-A, Yamazaki S, Tei H, Sakaki Y, Menaker M. Entrainment of the circadian clock in the liver by feeding. Science (80- ). 2001;291(5503):490–3. 10.1126/science.291.5503.490 11161204

[14] Welsh DK, Yoo S-H, Liu AC, Takahashi JS, Kay SA. Bioluminescence imaging of individual fibroblasts reveals persistent, independently phased circadian rhythms of clock gene expression. Curr Biol. 2004;14(24):2289–95. 15620658 10.1016/j.cub.2004.11.057 PMC3777438

[15] Nagoshi E, Saini C, Bauer C, Laroche T, Naef F, Schibler U. Circadian gene expression in individual fibroblasts. Cell. 2004;119(5):693–705. 15550250 10.1016/j.cell.2004.11.015

[16] Gray KM, Stroka KM. Vascular endothelial cell mechanosensing: new insights gained from biomimetic microfluidic models. Semin Cell Dev Biol. 2017;71:106–17. 28633977 10.1016/j.semcdb.2017.06.002

[17] Thompson CL, Fu S, Heywood HK, Knight MM, Thorpe SD. Mechanical stimulation: a crucial element of organ-on-chip models. Front Bioeng Biotechnol. 2020;10:8. 10.3389/fbioe.2020.602646 PMC7758201 33363131

[18] Cyr KJ, Avaldi OM, Wikswo JP. Circadian hormone control in a human-on-a-chip: *In vitro* biology’s ignored component? Exp Biol Med. 2017;242(17):1714–31. 10.1177/1535370217732766 PMC5832251 29065796

[19] Fustin JM, Li M, Gao B, Chen Q, Cheng T, Stewart AG. Rhythm on a chip: circadian entrainment in vitro is the next frontier in body-on-a chip technology. Curr Opin Pharmacol. 2019;48:127–36. 31600661 10.1016/j.coph.2019.09.005

[20] Jufri NF, Mohamedali A, Avolio A, Baker MS. Mechanical stretch: physiological and pathological implications for human vascular endothelial cells. Vasc Cell. 2015;7(1):8. 26388991 10.1186/s13221-015-0033-z PMC4575492

[21] Zhang EE, Liu AC, Hirota T, Miraglia LJ, Welch G, Pongsawakul PY, et al. A genome-wide rnai screen for modifiers of the circadian clock in human cells. Cell. 2009;139(1):199–210. 19765810 10.1016/j.cell.2009.08.031 PMC2777987

[22] Yang N, Williams J, Pekovic-Vaughan V, Wang P, Olabi S, McConnell J, et al. Cellular mechano-environment regulates the mammary circadian clock. Nat Commun. 2017;8(1):14287. 28134247 10.1038/ncomms14287 PMC5290282

[23] Heywood HK, Lee DA. Bioenergetic reprogramming of articular chondrocytes by exposure to exogenous and endogenous reactive oxygen species and its role in the anabolic response to low oxygen. J Tissue Eng Regen Med. 2017;11(8):2286–94. 26799635 10.1002/term.2126 PMC5172424

[24] Bleuel J, Zaucke F, Brüggemann G-P, Niehoff A. Effects of cyclic tensile strain on chondrocyte metabolism: a systematic review. PLoS ONE. 2015;10(3):e0119816. 25822615 10.1371/journal.pone.0119816 PMC4379081

[25] Thompson CL, McFie M, Chapple JP, Beales P, Knight MM. Polycystin-2 is required for chondrocyte mechanotransduction and traffics to the primary cilium in response to mechanical stimulation. Int J Mol Sci. 2021;22(9):4313. 33919210 10.3390/ijms22094313 PMC8122406

[26] Sachs M. Cosinor. Tools for estimating and predicting the cosinor model. R package. 2014. Available from: https://cran.r-project.org/package=cosinor. Accessed 30 Aug 2022.

[27] Jagannath A, Butler R, Godinho SIH, Couch Y, Brown LA, Vasudevan SR, et al. The CRTC1-SIK1 pathway regulates entrainment of the circadian clock. Cell. 2013;154(5):1100–11. 23993098 10.1016/j.cell.2013.08.004 PMC3898689

[28] Gossan N, Zeef L, Hensman J, Hughes A, Bateman JF, Rowley L, et al. The circadian clock in murine chondrocytes regulates genes controlling key aspects of cartilage homeostasis. Arthritis Rheum. 2013;65(9):2334–45. 23896777 10.1002/art.38035 PMC3888512

[29] Zhang R, Lahens NF, Ballance HI, Hughes ME, Hogenesch JB. A circadian gene expression atlas in mammals: Implications for biology and medicine. Proc Natl Acad Sci. 2014;111(45):16219–24. 25349387 10.1073/pnas.1408886111 PMC4234565

[30] Hermida RC, Ayala DE. Chronotherapy with the angiotensin-converting enzyme inhibitor ramipril in essential hypertension. Hypertension. 2009;54(1):40–6. 10.1161/HYPERTENSIONAHA.109.130203 19433778

[31] Ruben MD, Smith DF, FitzGerald GA, Hogenesch JB. Dosing time matters. Science. 2019;365(6453):547–9. 31395773 10.1126/science.aax7621 PMC8011856

[32] Dudek M, Yang N, Ruckshanthi JP, Williams J, Borysiewicz E, Wang P, et al. The intervertebral disc contains intrinsic circadian clocks that are regulated by age and cytokines and linked to degeneration. Ann Rheum Dis. 2017;76(3):576–84. 27489225 10.1136/annrheumdis-2016-209428 PMC5446006

[33] Soul J, Dunn SL, Anand S, Serracino-Inglott F, Schwartz J-M, Boot-Handford RP, et al. Stratification of knee osteoarthritis: two major patient subgroups identified by genome-wide expression analysis of articular cartilage. Ann Rheum Dis. 2018;77(3):423–423. 29273645 10.1136/annrheumdis-2017-212603 PMC5867416

[34] Fisch KM, Gamini R, Alvarez-Garcia O, Akagi R, Saito M, Muramatsu Y, et al. Identification of transcription factors responsible for dysregulated networks in human osteoarthritis cartilage by global gene expression analysis. Osteoarthr Cartil. 2018;26(11):1531–8. 10.1016/j.joca.2018.07.012 PMC6245598 30081074

[35] Cheng B, Anea CB, Yao L, Chen F, Patel V, Merloiu A, et al. Tissue-intrinsic dysfunction of circadian clock confers transplant arteriosclerosis. Proc Natl Acad Sci. 2011;108(41):17147–52. 21969583 10.1073/pnas.1112998108 PMC3193243

[36] Chowdhury TT, Bader DL, Shelton JC, Lee DA. Temporal regulation of chondrocyte metabolism in agarose constructs subjected to dynamic compression. Arch Biochem Biophys. 2003;417(1):105–11. 12921786 10.1016/s0003-9861(03)00340-0

[37] Yagita K, Okamura H. Forskolin induces circadian gene expression of rPer1, rPer2 and dbp in mammalian rat-1 fibroblasts. FEBS Lett. 2000;465(1):79–82. 10620710 10.1016/s0014-5793(99)01724-x

[38] Ginty DD, Kornhauser JM, Thompson MA, Bading H, Mayo KE, Takahashi JS, et al. Regulation of CREB Phosphorylation in the Suprachiasmatic Nucleus by Light and a Circadian Clock. Science. 1993;260(5105):238–41. 8097062 10.1126/science.8097062

[39] Tischkau SA, Mitchell JW, Tyan S-H, Buchanan GF, Gillette MU. Ca2+/cAMP response element-binding protein (creb)-dependent activation of per1 is required for light-induced signaling in the suprachiasmatic nucleus circadian clock. J Biol Chem. 2003;278(2):718–23. 12409294 10.1074/jbc.M209241200

[40] Lee B, Aiqing Li, Hansen KF, Ruifeng Cao, Jae Hwa Yoon, Obrietan K. CREB Influences timing and entrainment of the SCN circadian clock. J Biol Rhythms. 2010;25(6):410–20. 10.1177/0748730410381229 PMC3529591 21135157

[41] Gau D, Lemberger T, von Gall C, Kretz O, Le Minh N, Gass P, et al. Phosphorylation of CREB Ser142 regulates light-induced phase shifts of the circadian clock. Neuron. 2002;34(2):245–53. 11970866 10.1016/s0896-6273(02)00656-6

[42] O’Neill JS, Maywood ES, Chesham JE, Takahashi JS, Hastings MH. cAMP-dependent signaling as a core component of the mammalian circadian pacemaker. Science. 2008;320(5878):949–53. 18487196 10.1126/science.1152506 PMC2735813

[43] Ogawa H, Kozhemyakina E, Hung H-H, Grodzinsky AJ, Lassar AB. Mechanical motion promotes expression of Prg4 in articular cartilage via multiple CREB-dependent, fluid flow shear stress-induced signaling pathways. Genes Dev. 2014;28(2):127–39. 24449269 10.1101/gad.231969.113 PMC3909787

