# Supplementary material for: Oscillations of the circadian clock protein, BMAL-1, align to daily cycles of mechanical stimuli: a novel means to integrate biological time within predictive in vitro model systems
Source: In Vitro Model. 2022 Aug 31;1(6):405–12. doi: 10.1007/s44164-022-00032-x (PMC9767245; doi:10.1007/s44164-022-00032-x)
Supplement: Supplementary file 1 — Supplementary file1 (PDF 1059 KB) [file 44164_2022_32_MOESM1_ESM.pdf]

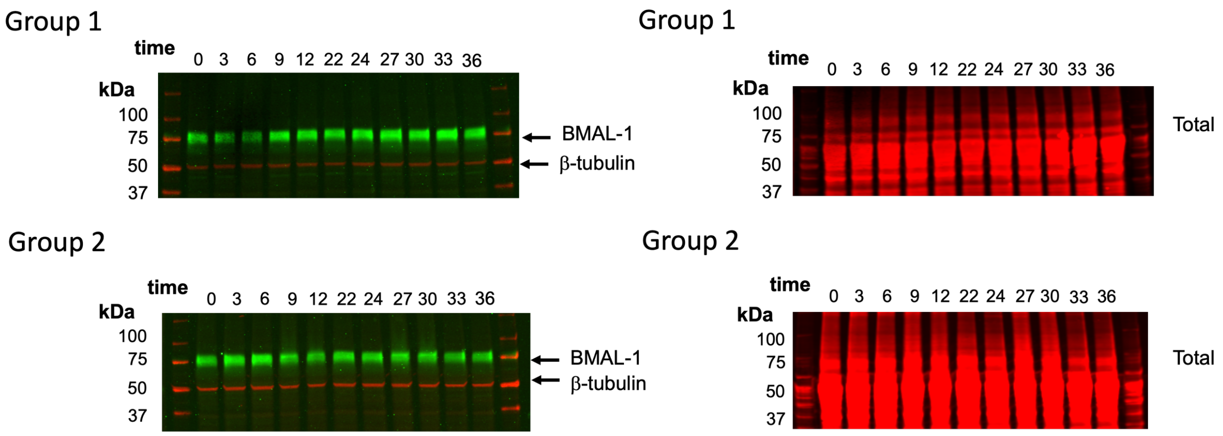

Figure S1. **Representative Western Blots illustrating BMAL-1 abundance (Left) and total protein (Right) in 36 h timeseries during constant unloaded conditions following entrainment by mechanical stimuli.** Protein samples from Group 1 (Top) and Group 2 (bottom) were collected in parallel during constant unloaded conditions following a 3-day entrainment period. Equal amounts of protein were loaded into each lane of mini-protean TGX 4-20% gels (Biorad), with one gel for each timeseries. Molecular weight markers (mw) are in first and last lanes. After protein transfer onto nitrocellulose membranes (Biorad), total protein was quantified using LiCor Total Protein Stain, shown on the right. Anti-BMAL-1 antibody (abcam ab93806) and secondary antibody (Licor, 925-32213) reveal BMAL-1 protein at ~75 kDa, (green), shown on the left. β-Tubulin is indicated at ~50 kDa (red). The 800 nm fluorescence signal from BMAL-1 was quantified using the Image Studio Lite software (Li-Cor Bioscience) and normalised to total protein (700 nm).
